# Proteomics of prostate cancer – revealing how cancer cells master their messy genomes

**DOI:** 10.18632/oncoscience.453

**Published:** 2018-07-31

**Authors:** Leena Latonen, Matti Nykter, Tapio Visakorpi

**Affiliations:** Leena Latonen: Prostate Cancer Research Center, Faculty of Medicine and Life Sciences and BioMediTech Institute, University of Tampere, Tampere, Finland; FimLab laboratories, Tampere University Hospital, Tampere, Finland

**Keywords:** prostate cancer, proteomics, proteogenomics, castration resistance

Recent sequencing endeavours have manifested the surprising magnitude and variation in prostate cancer mutations, warranting for data of the output of all these aberrations at the proteomic and functional level. We performed a large-scale proteomic analysis of a cohort of clinical prostate cancer samples [1]. With integration of the proteomic data to genetic, epigenetic and transcriptomic data from the same specimens, we could ask the question of how much of the genomic and transcriptomic aberrations are reflected at the protein level. Our results showed that prostate cancer can buffer out a large fraction of the accrued changes during disease progression, most likely to maintain intact core functions to survive and thrive. With proteomics, we perceive how the cancer cells make sense out of the genomic chaos within them.

The plethora of genetic and transcriptomic changes especially in castration resistant prostate cancer (CRPC) have become astoundingly clear with extensive research during the past decades. Especially, the consortium sequencing efforts such as TCGA and SU2C have been able to reveal the extent of heterogeneity of prostate cancer mutations [2, 3]. Considering the “long tail of driver mutations” in prostate cancer [3], the personalized medicine approach based on targeting individual mutations with designer drugs starts to stagger in its auspiciousness. As the effects of most aberrations harboured in DNA and RNA flow through proteins, understanding the translations of cancer genomes should help us focus on the right parts of the picture.

The cancer field is awakening to this challenge. For example, The National Cancer Institute’s Clinical Proteomic Tumor Analysis Consortium (CPTAC) was launched to supplement TCGA data with mass spectrometry-based proteomics [4]. In parallel, bioinformatics community has acknowledged the need for more in-depth understanding and more advanced computational methods to study relationships between transcriptomes and proteomes. To advance this, a proteogenomic DREAM challenge was proposed [5], where the goal is to predict protein expression or phosphorylation levels from genomic and transcriptomic data types. Data from the above-mentioned CPTAC is then used to evaluate and rank the prediction methods.

Our approach included unlabeled high throughput mass spectrometry on clinical tissue samples of untreated primary prostate cancer (PC), CRPC and benign prostatic hyperplasia (BPH) [1]. We demonstrated that each of these groups shows a distinct protein profile, and that the proteomic status of prostate tissue undergoes considerable changes during cancer development and progression.

Strikingly, in our integrative analysis we showed that gene copy number, DNA methylation, and RNA expression levels do not reliably predict these proteomic changes. Neither the altered gene dosages, nor the global methylation changes were translated to the level of the proteome to the same extent as they are correlated with the global RNA expression. This was especially prominent in CRPC that has accrued significantly more genomic aberrations than primary cancers. Thus, a large proportion of these changes in the progressed tumors seem as side products of the catastrophic state of the genome. As they are untranslated, they are, subsequently, left without a functional effect at the protein level.

A marked leap forward with large scale proteomic data was to identify several miRNA - target correlations present at protein but not at mRNA level. miRNA binding does not always lead to degradation of the target mRNA, allowing us to identify dozens of potentially relevant pairs that were not revealed by transcriptomic data only. As these correlations need to be validated for each individual pair, we already verified several pairs, thus giving promise to further true events to be found based on our resource.

The proteomics provided novel pathway alterations in prostate cancer. Especially, we identified two metabolic shifts in the citric acid cycle (TCA cycle) during prostate cancer development and progression. AR, the key promoter of castration resistance, functions primarily as a transcription factor, and has been indicated to regulate also prostate cancer metabolism [6]. In consistence with the likely posttranslational nature of the regulatory events we identified, we found no evidence of the TCA enzymes being transcriptionally androgen regulated (data not shown) [7]. Thus, the mechanisms behind the sequential metabolic shifts warrant further investigation.

Mere presence of a protein does not necessarily indicate activity, making large-scale assessment of PTMs also crucial. An analysis of genomic and transcriptomic data combined with phosphoproteomics provided a valuable view on active signaling pathways in CRPC [8]. A future aim of the field must be to combine the full proteogenomic view with the active signalome. Furthermore, large scale metabolomics would add to a comprehensive view of prostate cancer alterations.

As the most common male malignancy in Western countries, and the second most common cancer among men overall, prostate cancer is a heavy burden to society. Many of these cancers are indolent in nature, not requiring active treatment. However, a substantial fraction of the cancers are potentially lethal. Thus, biomarkers that identify the aggressive form of the disease are urgently needed. In addition, without curative treatment against CRPC, there is a pressing need to understand the disease development and progression better in order to find better therapies. The proteomic view that we provided, which will undoubtedly be supplemented by further studies, emphasizes how important it is to inspect the cancer changes at the functional level. This way we can focus our future efforts on the hub factors and key functions. Our study already revealed novel mechanisms with potential for future therapeutic targeting, as well as provided potential novel diagnostic and therapeutic markers.
